# Dietary shredded steam-exploded pine particle supplementation as a strategy to mitigate chronic cyclic heat stress by modulating gut microbiota in broilers

**DOI:** 10.1038/s41598-022-24031-w

**Published:** 2022-11-16

**Authors:** Akshat Goel, Chris Major Ncho, Beom-June Kim, Chae-Mi Jeong, Vaishali Gupta, Ji-Young Jung, Si-Young Ha, Jae-Kyung Yang, Yang-Ho Choi

**Affiliations:** 1 Department of Animal Science, Gyeongsang National University, Jinju, 52828 Korea; 2 Institute of Agriculture and Life Sciences, Gyeongsang National University, Jinju, 52828 Korea; 3 Division of Applied Life Sciences (BK21 Plus Program), Gyeongsang National University, Jinju, 52828 Korea; 4 Department of Environmental Materials Science, Gyeongsang National University, Jinju, 52828 Korea

**Keywords:** Environmental impact, Applied microbiology, Animal biotechnology, Animal behaviour, Animal physiology, Biochemistry

## Abstract

Improving the availability of underutilized waste for the economic use of livestock feed can be important in countries where feed grain production is scarce. Modulating the gut microbiota through the fibrous content present in these wastes may help mitigate the adverse effects of heat stress (HS). Here, we investigated the effects of dietary steam-exploded pine particle (SPP), a value-added waste product, on the performance, gut health, and cecum microbiota in heat-stressed broilers. Ross 308 broilers (n = 180) at 29 days of age were distributed into three dietary treatment groups (0%, 1%, and 2% SPP) and two temperature conditions (NT: 21 °C; CHS: 31 °C) and grown for seven days. CHS, but not SPP, adversely affected performance parameters, but SPP did not interactively modulate these results. On the contrary, both differently affected other parameters. CHS resulted in increased rectal temperature, total protein in serum, and Nox4 gene expression, whereas 2% SPP increased GLP-2 and the Nox4 gene expression in the duodenum in comparison to 0% and 1% SPP. CHS significantly modified the beta-diversity of cecal microbiota while 1% SPP supplementation in diets increased the abundance of the favorable bacterial genera in chicken. Concludingly, CHS adversely affects growth performances, gut health, stress-related genes, and cecal microbiota while dietary 1% SPP may facilitate the proliferation of beneficial microorganisms in the cecum of broilers.

## Introduction

An increase in environmental temperature is one of the common impressions of global warming. Occurrences of the extended summer season in most countries lead to a direct effect on livestock animals. Poultry is one of those animals facing severe production losses due to high ambient temperatures^1^. Heat stress (HS) can negatively affect growth performance, immunity parameters, and mortality rate in chickens^2,3^.

Dietary supplementation of various additives could help overcome these deleterious effects. Feed contributes the major portion of expenditure for rearing chickens. The extensive competitive nature of the poultry industry demands suitable additives to keep the price at the lower end. Their importance increases significantly in countries that rely on imports of key feed ingredients. Therefore, cheap ingredients of low economic importance should be explored for inclusion in feed.

Wood powder is among the most inexpensive materials available in the form of byproducts from woodworking operations such as sawing, sanding, milling, and routing. Cellulose and lignin are the main constituents of wood powder that may vary according to the source and type of wood materials^4^. Furthermore, the wood powder is rich in insoluble fiber content^5^. Recent technology advancements have helped modulate the insoluble fiber content by depolymerizing the hemicellulose of the wood powder through the steam explosion method^6,7^. The major benefits of such products could be through their prebiotic effects, acting as a substrate to promote beneficial bacteria and retarding the pathogenic bacteria present in the intestine^8,9^. Prebiotic products can modulate the cecum microbiota in heat exposed-broilers^10^. Supplementing chicken feed with chopped steam-blasted pine particles may increase the abundance of fibrinolytic bacteria in the cecum without adverse effects^11^. Furthermore, phenols from lignin can modulate broiler production and health by enhancing the CD4+ and CD8+ lymphocytes in the duodenum^12,13^.

Supplementation of a modified form of wood powder after a steam explosion may help reduce the adverse effect of HS on gut health by promoting the antimicrobial, immunomodulatory, and growth-promoting properties of chicken. Several experiments have been conducted to evaluate the effect of different dietary supplements on HS in chickens. However, limited information is available on the use of wood powder as a dietary supplement in chicken feed. The easy and cheap availability of wood powder encouraged us to use it as a prebiotic product in poultry diets. Furthermore, a gradual increase in the environmental temperature during the daytime in commercial animal houses made us select the cyclic form of HS to consider for thermal exposure. In this study, we determined the effect of dietary supplementation of increasing the concentration of shredded steam-exploded pine particle (SPP) on the growth performances, gut health, and modulation in cecal microbiota in cyclic heat-stressed (CHS) broilers during the final week of age near slaughter.

## Results

### Growth performance

The effect of different doses of SPP on the growth performance parameters of birds kept at either NT or CHS has been presented in Table 1. Before the start of the experiment, the initial body weight was similar among all the treatment groups. The final body weight taken after seven days of the experiment indicated no dose or interaction effects. However, in terms of temperature effects, there was a nonsignificant decrease (P = 0. 090) in the body weights of birds exposed to CHS in comparison to NT. Furthermore, the percent difference in body weight has shown a temperature effect and had a lower weight (P = 0.001) in CHS in comparison to NT. No dose or interaction effects were observed in terms of a percent difference in body weight. Growth parameters such as ADG, ADFI, and FCR have shown a temperature effect. The ADG and ADFI were lower (P = 0.001 and P = 0.021) while FCR was higher (P = 0.001) in CHS birds in comparison to NT. No interaction or dose effect was noticed in ADG, ADFI, and FCR of chicken fed with increasing concentration of SPP in diets. No mortality was recorded during the HS period of the entire experiment. All birds were found healthy and showed normal behavior until the end of the experiment.

**Table 1:** Effect of dietary steam-exploded pine particle supplementation on the growth performances of broilers reared under normal or cyclic heat stress conditions.

		Initial bwt (day 28)	Final bwt (day 35)	% difference in bwt	ADG (28–35)	ADFI (28–35)	FCR (28–35)
0%	NT	1463 ± 54	2154 ± 75	47.3 ± 1.1	98.7 ± 3.6	170.0 ± 5.5	1.72 ± 0.01
	HS	1513 ± 33	2143 ± 30	41.9 ± 2.1	90.0 ± 3.4	167.3 ± 2.2	1.82 ± 0.04
1%	NT	1483 ± 34	2231 ± 62	50.4 ± 1.9	106.8 ± 5.0	176.7 ± 5.6	1.66 ± 0.03
	HS	1479 ± 50	2129 ± 57	44.2 ± 2.4	92.9 ± 3.6	165.8 ± 4.3	1.79 ± 0.04
2%	NT	1543 ± 34	2295 ± 57	48.7 ± 0.8	107.4 ± 3.5	179.4 ± 4.9	1.67 ± 0.02
	HS	1499 ± 39	2164 ± 49	44.5 ± 0.9	95.1 ± 1.8	166.8 ± 2.7	1.75 ± 0.01
**Main effects**							
Temp	NT	1496 ± 24	2227 ± 38	48.8 ± 0.8	104.3 ± 2.4	175.4 ± 3.0	1.69 ± 0.01
	HS	1497 ± 23	2145 ± 26	43.5 ± 1.1	92.7 ± 1.7	166.6 ± 1.7	1.79 ± 0.02
Dose	0%	1488 ± 31	2148 ± 39	44.6 ± 1.4	94.4 ± 2.7	168.7 ± 2.8	1.77 ± 0.02
	1%	1481 ± 29	2180 ± 43	47.3 ± 1.8	99.8 ± 3.6	171.3 ± 3.7	1.73 ± 0.03
	2%	1521 ± 25	2229 ± 41	46.6 ± 0.8	101.2 ± 2.6	173.1 ± 3.3	1.71 ± 0.02
**P-value**							
Temp		0.991	0.090	0.001	0.001	0.021	0.001
Dose		0.595	0.367	0.257	0.150	0.606	0.107
Temp × dose		0.532	0.553	0.843	0.755	0.491	0.633

*bwt* body weight, *NT* thermoneutral temperature, *HS* heat stress, *ADG* average daily gain, *ADF*I average daily feed intake, *FCR* feed conversion ratio.

Values are presented as mean with standard error (n = 6).

### Rectal temperature

The RT was recorded before and after the HS on the final day of the experiment (Table 2). No variation was noticed in the RT of birds when taken before the HS on the final day of the experiment. After seven days of CHS, temperature effects (P = 0.001) were observed and higher RT was recorded in CHS in comparison to birds kept at NT.

**Table 2:** Effect of dietary steam-exploded pine particle supplementation on the rectal temperatures (°C) of broilers reared under normal or cyclic heat stress conditions.

		RT before	RT after
0%	NT	42.04 ± 0.08	41.71 ± 0.03
	HS	42.00 ± 0.06	42.83 ± 0.07
1%	NT	42.00 ± 0.11	41.73 ± 0.05
	HS	41.87 ± 0.06	42.70 ± 0.05
2%	NT	41.71 ± 0.09	41.73 ± 0.04
	HS	41.94 ± 0.06	42.96 ± 0.06
**Main effects**			
Temp	NT	41.92 ± 0.06	41.72 ± 0.02
	HS	41.94 ± 0.03	42.83 ± 0.04
Dose	0%	42.02 ± 0.05	42.27 ± 0.16
	1%	41.94 ± 0.06	42.21 ± 0.14
	2%	41.83 ± 0.06	42.34 ± 0.17
**P-value**			
Temp		0.767	0.001
Dose		0.059	0.060
Temp × dose		0.071	0.060

*RT* rectal temperature, *NT* thermoneutral temperature, *HS* heat stress.

Values are presented as mean with standard error (n = 6).

### Organ length and weight

The effect of dietary SPP supplementation at NT and CHS on the organ length is presented in Table 3. No temperature, dose, or interaction effects were observed in the different portions of the intestinal length of broilers.

**Table 3:** Effect of dietary steam-exploded pine particle supplementation on the organ length (cm/100gm body weight) of broilers reared under normal or cyclic heat stress conditions.

		Duodenum	Jejunum	Ileum	Cecum
0%	NT	1.32 ± 0.04	3.15 ± 0.17	3.13 ± 0.15	0.83 ± 0.04
	HS	1.34 ± 0.06	3.23 ± 0.10	2.95 ± 0.11	0.80 ± 0.02
1%	NT	1.26 ± 0.05	3.07 ± 0.12	2.96 ± 0.07	0.77 ± 0.02
	HS	1.34 ± 0.05	3.30 ± 0.12	3.12 ± 0.08	0.84 ± 0.03
2%	NT	1.25 ± 0.05	3.02 ± 0.08	2.92 ± 0.10	0.83 ± 0.04
	HS	1.25 ± 0.04	3.08 ± 0.13	3.00 ± 0.14	0.81 ± 0.05
**Main effects**					
Temp	NT	1.28 ± 0.03	3.08 ± 0.07	3.01 ± 0.06	0.81 ± 0.02
	HS	1.31 ± 0.03	3.21 ± 0.07	3.02 ± 0.06	0.82 ± 0.02
Dose	0%	1.33 ± 0.04	3.19 ± 0.10	3.04 ± 0.09	0.81 ± 0.02
	1%	1.30 ± 0.03	3.19 ± 0.09	3.04 ± 0.06	0.81 ± 0.02
	2%	1.25 ± 0.03	3.05 ± 0.07	2.96 ± 0.08	0.82 ± 0.03
**P-value**					
Temp		0.407	0.218	0.843	0.76
Dose		0.262	0.433	0.699	0.896
Temp × dose		0.620	0.750	0.303	0.237

*NT* thermoneutral temperature, *HS* heat stress.

Values are presented as mean with standard error (n = 7).

The relative organ weight is presented in Table 4. No temperature, dose, or interaction effects were observed on the weight of duodenum, jejunum, ileum, and liver in broilers except duodenum had interaction effects (P = 0.047). Temperature effects (P = 0.022) were observed on the spleen weight of the broiler indicating lower splenic weight in CHS-exposed birds. No dose or interaction effect was observed on the weight of the spleen in broilers.

**Table 4:** Effect of dietary steam-exploded pine particle supplementation on the relative organ weight (percent body weight) of broilers reared under normal or cyclic heat stress conditions.

		Duodenum	Jejunum	Ileum	Liver	Spleen
0%	NT	0.45 ± 0.02	0.95 ± 0.06	0.77 ± 0.05	2.43 ± 0.12	0.14 ± 0.03
	HS	0.40 ± 0.02	0.89 ± 0.05	0.74 ± 0.07	2.33 ± 0.08	0.12 ± 0.01
1%	NT	0.41 ± 0.01	0.98 ± 0.04	0.78 ± 0.03	2.39 ± 0.06	0.16 ± 0.02
	HS	0.41 ± 0.02	0.93 ± 0.04	0.83 ± 0.04	2.31 ± 0.13	0.11 ± 0.01
2%	NT	0.39 ± 0.01	0.92 ± 0.02	0.76 ± 0.03	2.19 ± 0.07	0.13 ± 0.02
	HS	0.46 ± 0.04	0.96 ± 0.03	0.75 ± 0.03	2.33 ± 0.11	0.11 ± 0.01
**Main effects**						
Temp	NT	0.42 ± 0.01	0.95 ± 0.02	0.77 ± 0.02	2.34 ± 0.05	0.14 ± 0.01
	HS	0.42 ± 0.02	0.93 ± 0.02	0.77 ± 0.03	2.32 ± 0.06	0.11 ± 0.01
Dose	0%	0.43 ± 0.02	0.92 ± 0.04	0.75 ± 0.04	2.38 ± 0.07	0.13 ± 0.01
	1%	0.41 ± 0.01	0.95 ± 0.03	0.81 ± 0.02	2.35 ± 0.07	0.14 ± 0.01
	2%	0.43 ± 0.02	0.94 ± 0.02	0.75 ± 0.02	2.26 ± 0.07	0.12 ± 0.01
**P-value**						
Temp		0.733	0.576	0.917	0.878	0.022
Dose		0.733	0.761	0.325	0.442	0.553
Temp × dose		0.047	0.437	0.566	0.405	0.719

*NT* thermoneutral temperature, *HS* heat stress.

Values are presented as mean with standard error (n = 7).

### Plasma metabolites

The effect of dietary SPP supplementation at NT and CHS on the plasma metabolites is presented in Table 5. No temperature, dose, or interaction effects were observed in the plasma glucose, triglyceride, and cholesterol levels of broilers. Temperature effects (P = 0.001) were observed on the plasma total protein of the broiler indicating an increase in the total protein levels in CHS exposed birds.

**Table 5:** Effect of dietary steam-exploded pine particle supplementation on the plasma metabolites of broilers reared under normal or cyclic heat stress conditions.

		Glucose	Total Protein	Triglyceride	Cholesterol
0%	NT	259.0 ± 16.7	2.9 ± 0.1	33.3 ± 3.8	115.4 ± 4.2
	HS	227.6 ± 4.9	3.3 ± 0.1	30.6 ± 6.9	122.3 ± 5.1
1%	NT	253.7 ± 10.3	2.8 ± 0.1	32.4 ± 2.6	119.6 ± 7.1
	HS	243.6 ± 6.4	3.2 ± 0.1	31.6 ± 5.6	124.6 ± 1.7
2%	NT	227.3 ± 6.5	2.6 ± 0.1	22.1 ± 2.9	116.3 ± 5.2
	HS	236.7 ± 4.8	3.1 ± 0.2	27.3 ± 3.5	122.1 ± 4.0
**Main effects**					
Temp	NT	246.7 ± 7.2	2.7 ± 0.1	29.3 ± 2.1	117.1 ± 3.1
	HS	236.0 ± 3.3	3.2 ± 0.1	29.8 ± 3.0	123.0 ± 2.1
Dose	0%	243.3 ± 9.4	3.1 ± 0.1	31.9 ± 3.8	118.9 ± 3.3
	1%	248.6 ± 6.0	3.0 ± 0.1	32.0 ± 3.0	122.1 ± 3.6
	2%	232.0 ± 4.1	2.8 ± 0.1	24.7 ± 2.3	119.2 ± 3.2
**P-value**					
Temp		0.164	0.001	0.887	0.143
Dose		0.199	0.082	0.187	0.768
Temp × dose		0.101	0.887	0.659	0.982

*NT* thermoneutral temperature, *HS* heat stress, *SEM* standard error of the mean.

Values are presented as mean with standard error (n = 7).

### Gut health and stress-related genes

**Figure 1:**
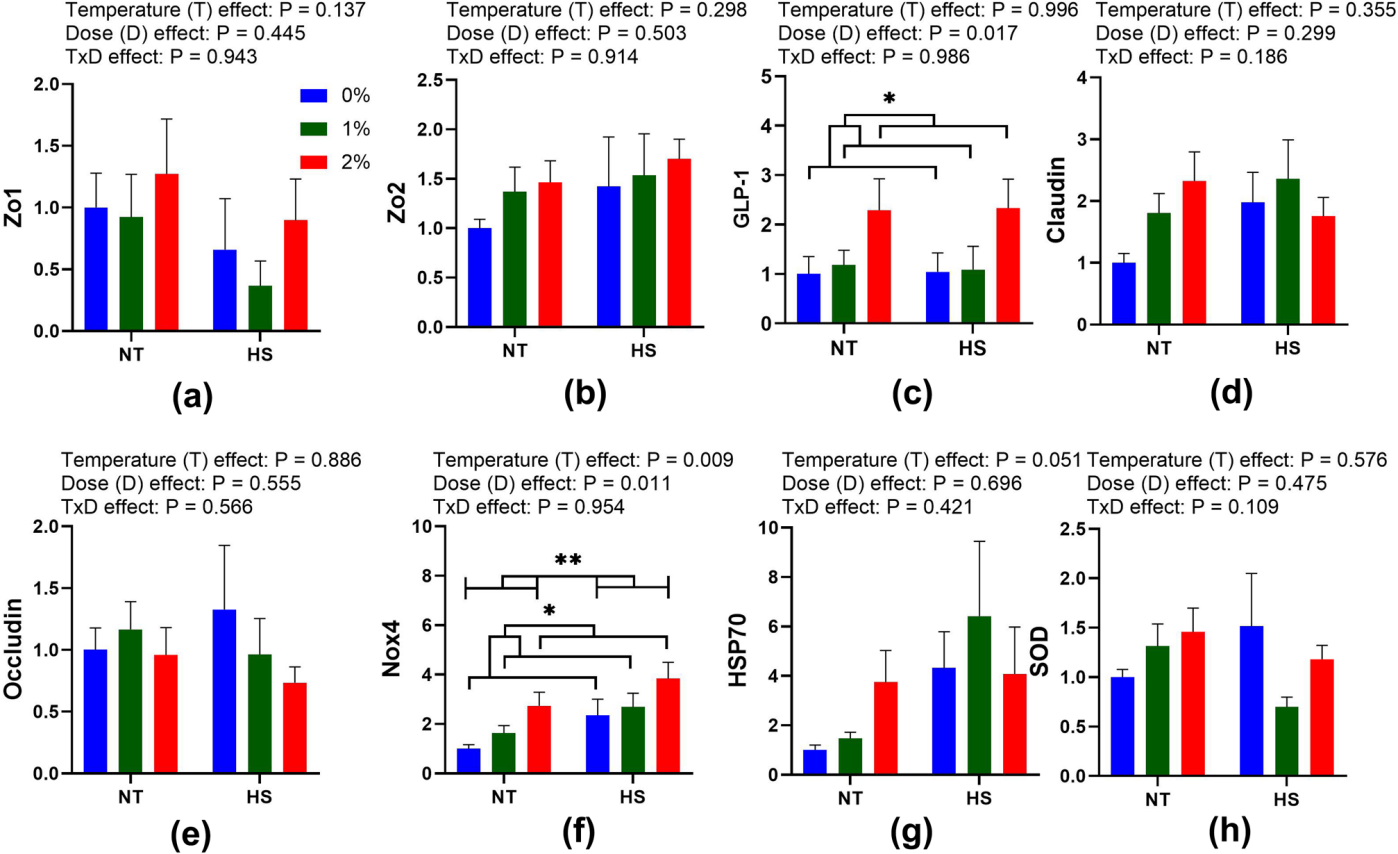
Effect of dietary steam-exploded pine particle supplementation on the gene expression profiling of genes (**a**) Zo1 (**b**) Zo2 (**c**) GLP-2 (**d**) Claudin (**e**) Occludin (**f**) Nox4 (**g**) Hsp70 (**h**) and SOD respectively, in the duodenum of broilers reared under normal or cyclic heat stress conditions. Dietary treatments were 0, 1, and 2% SPP in diets respectively. The temperature of the rooms was maintained at either thermoneutral (NT; 21 °C) or cyclic heat stress (CHS; 31 °C; 6 h daily) for seven days.

The effect of dietary SPP supplementation at NT and CHS on the expression of gut health-related genes (Zo1, Zo2, GLP-2, Claudin, and Occludin) and stress markers (NOX4, HSP70, and SOD) in the duodenum of broiler chickens is presented in Fig. 1 and Supplementary Table 3. No temperature, dose, or interaction effects were observed in the gene expression of Zo1, Zo2, Claudin-1, Occludin, HSP70, and SOD gene of broilers. The expression of GLP-2 (P = 0.017) and Nox4 (P = 0.011) genes has shown dose–effect and was found to be higher in 2% SPP supplemented diets in comparison to control and 1% SPP fed chickens. The expression of the Nox4 gene has also shown temperature effects (P = 0.009) and was higher in CHS exposed birds. Contrast analysis revealed higher expression of Claudin-1 gene in 2% NT (P = 0.037) and 1% HS (P = 0.027) in comparison to chicken fed with control diets at thermoneutral temperature (Supplementary Table 3). The results of the contrast analysis also suggested that the expression of HSP70 (P = 0.030) and SOD (P = 0.037) genes was higher in 1% HS in comparison to 0% NT and 0% HS, respectively (Supplementary Table 3).

### Alpha and beta-diversity of cecal microbiota

**Figure 2:**
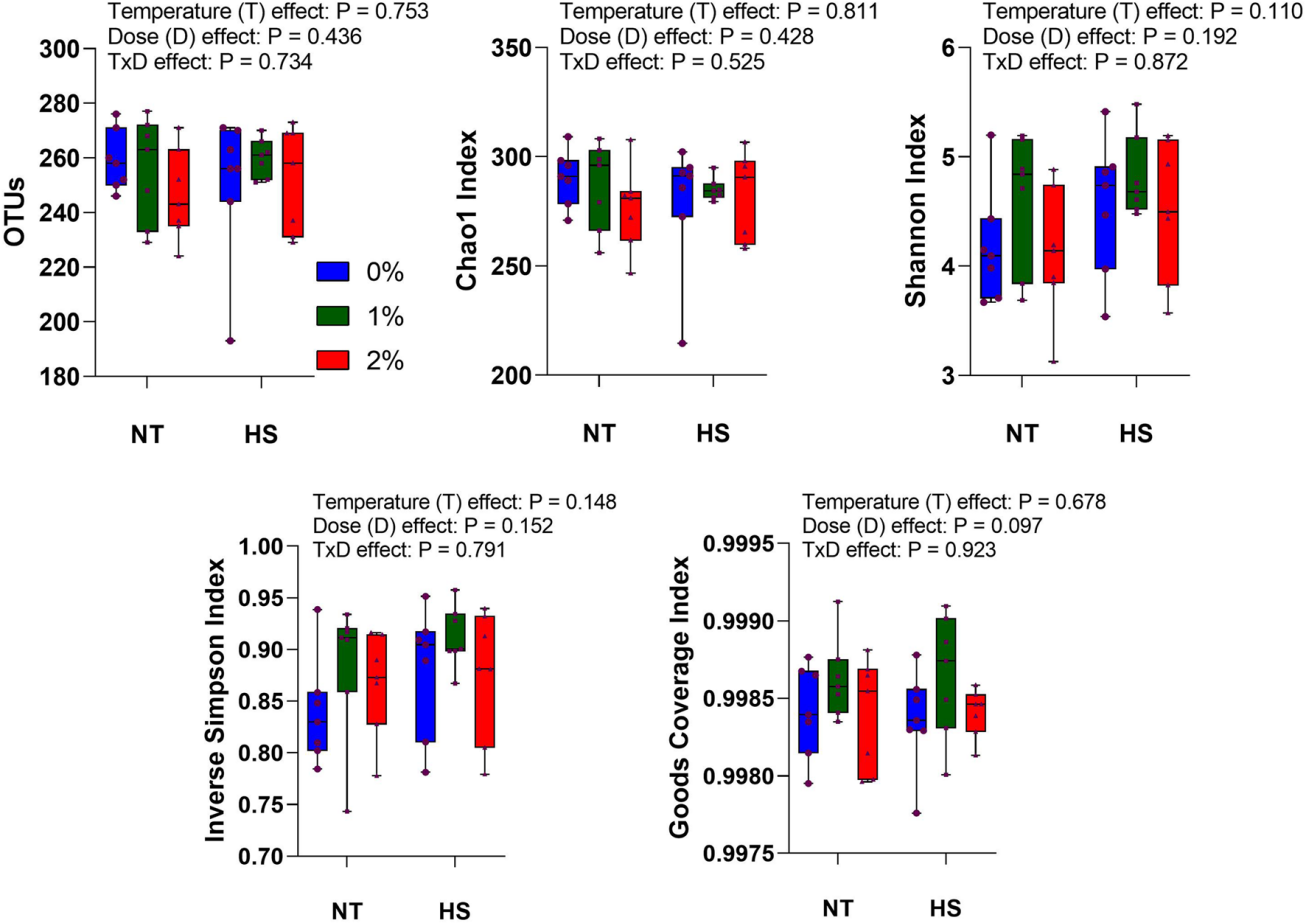
Effect of dietary steam-exploded pine particle supplementation on the community richness and alpha-diversity of cecum microflora of broilers reared under normal or cyclic heat stress conditions. Dietary treatments were 0, 1, and 2% SPP in diets respectively. The temperature of the rooms was maintained at either thermoneutral (NT; 21 °C) or cyclic heat stress (CHS; 31 °C; 6 h daily) for seven days.

To determine the alpha-diversity, community richness was analyzed through OTUs and Chao1, while diversity was analyzed using Shannon, Inverse Simpson, and Goods coverage indices (Fig. 2). No temperature, dose, or interaction effects (P > 0.05) were observed in the alpha-diversity indices.

**Figure 3:**
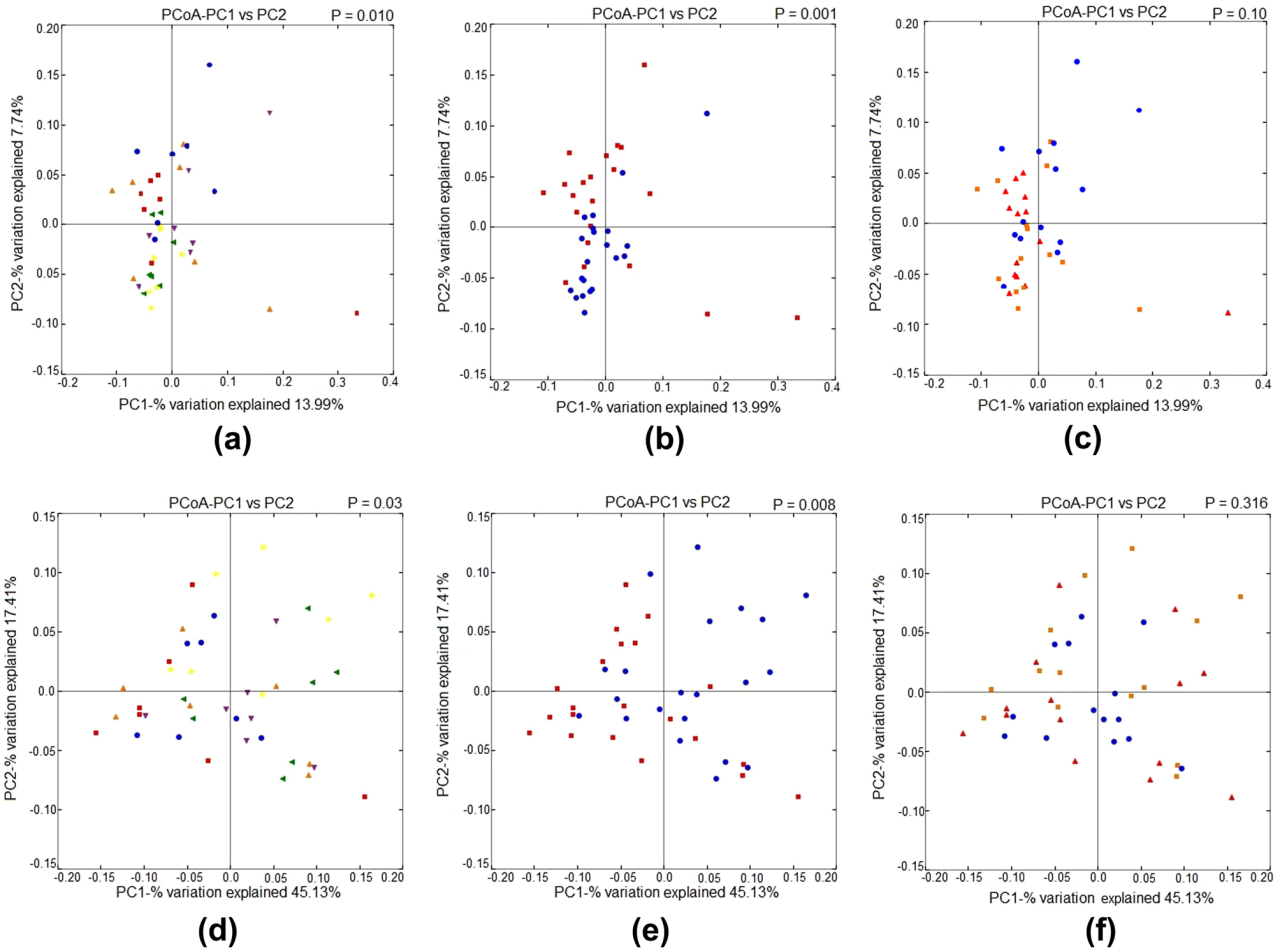
Effect of dietary steam-exploded pine particle supplementation on the beta-diversity of broilers reared under normal or cyclic heat stress conditions. Dietary treatments were 0, 1, and 2% SPP in diets respectively. The temperature of the rooms was maintained at either thermoneutral (NT; 21 °C) or cyclic heat stress (CHS; 31 °C; 6 h daily) for 7 days. Change in cecum microbiota based on unweighted and weighted UniFrac distances indicating interaction effect (**a** and **d**; green triangle indicates 0% NT, purple triangle indicates 1% NT and yellow triangle indicates 2% NT, red square indicates 0% HS, a blue circle indicates 1% HS and the orange triangle indicates 2% HS), heat stress effect (**b** and **e**; the blue circle indicates NT and red square indicates HS), dose–effect (**c** and **f**; red triangle indicates 0%, a blue circle indicates 1% and orange square indicates 2% dose of SPP) respectively.

To determine the beta-diversity, unweighted and weighted UniFrac distances were measured and presented in Fig. 3. The unweighted and weighted UniFrac distances based on PCoA revealed an interaction effect (PERMANOVA analysis, P = 0.01 and P = 0.001, respectively) and temperature effect (PERMANOVA analysis, P = 0.03 and P = 0.008, respectively). No dose–effect was observed in the unweighted and weighted UniFrac distances.

### Cecal microbial composition

**Figure 4:**
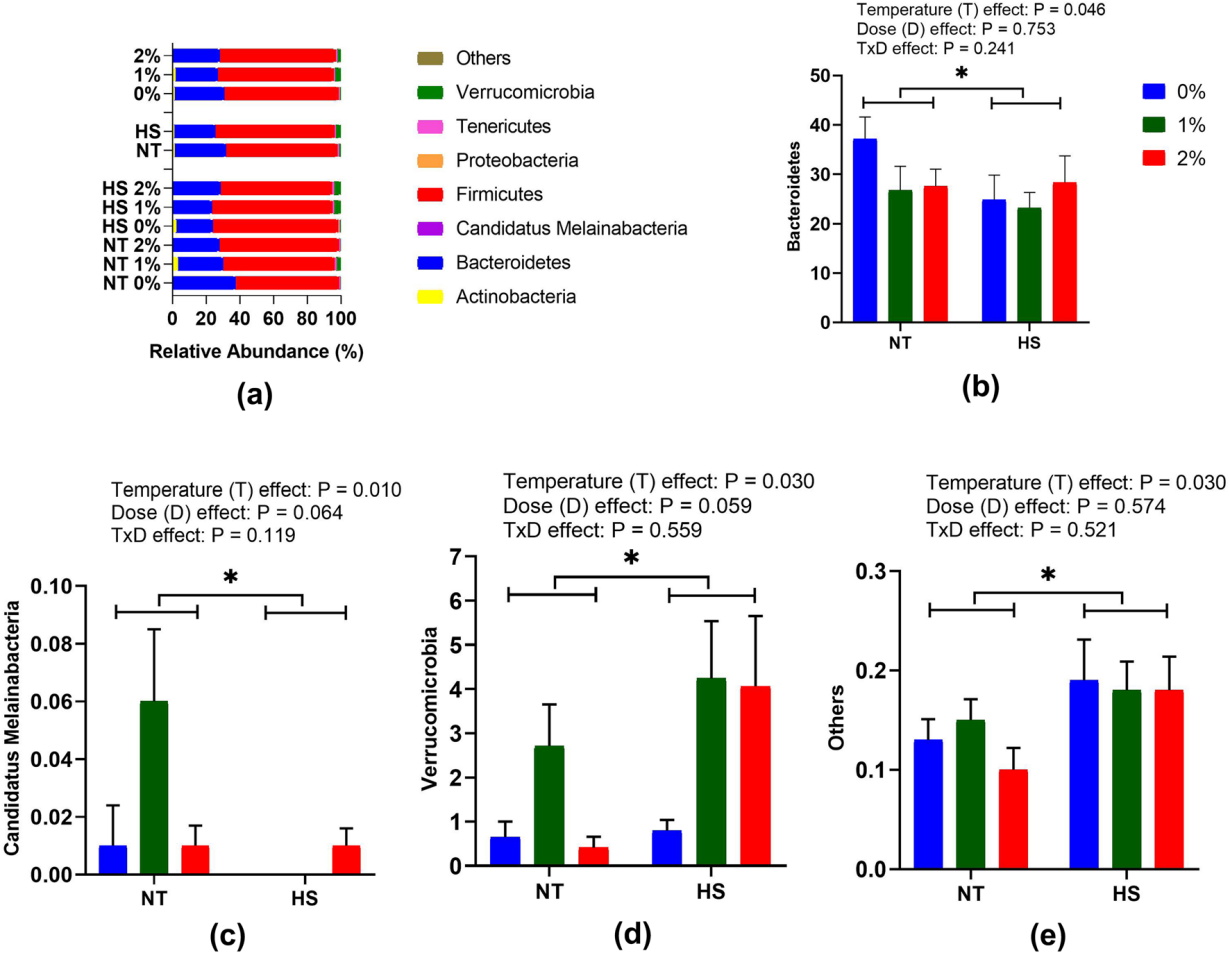
Effect of dietary steam-exploded pine particle supplementation on the relative abundance of phylum (**a**) and significant abundance microbiota namely Bacteroidetes (**b**) Candidatus Melainabacteria (**c**) Verrcomicrobia (**d**) and others respectively, in the cecum of broilers reared under normal or cyclic heat stress conditions. Dietary treatments were 0, 1, and 2% SPP in diets respectively. The temperature of the rooms was maintained at either thermoneutral (NT; 21 °C) or cyclic heat stress (CHS; 31 °C; 6 h daily) for seven days.

The effect of dietary SPP supplementation at NT and CHS on the cecal microbiota composition at the phylum level is presented in Fig. 4. Firmicutes were the major dominant phyla followed by Bacteroidetes. The abundance of Bacteroidetes (P = 0.046) and Candidatus Melainabacteria (P = 0.010) were lower while that of Verrucomicrobia (P = 0.030) and others (P = 0.030) were higher in CHS indicating temperature effect. No dose or interaction effects were observed in the cecal microbiota composition at the phylum level of broilers.

**Figure 5:**
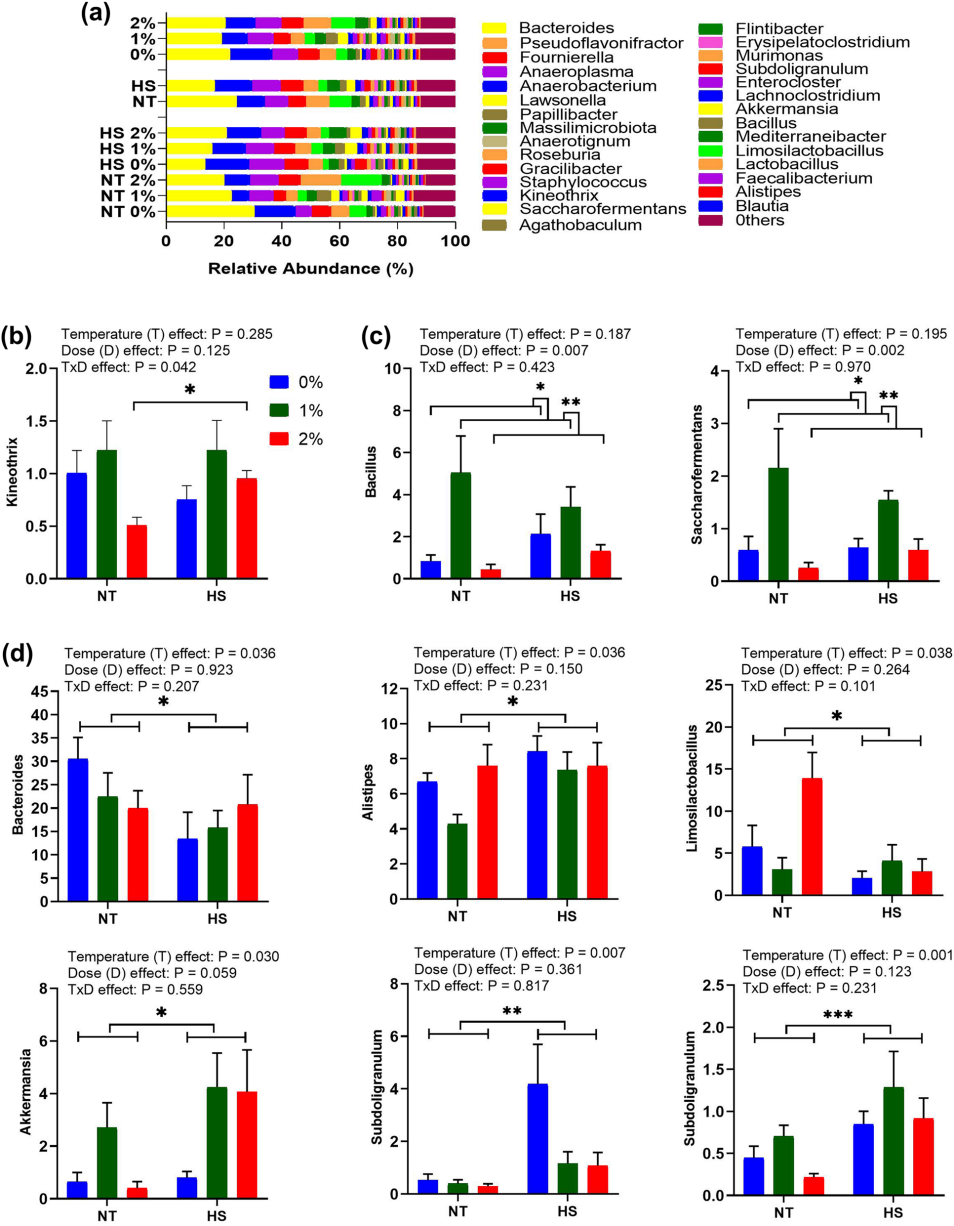
Effect of dietary steam-exploded pine particle supplementation on the relative abundance of top thirty most abundant genus (**a**) significantly abundance microbiota depicting interaction effect (**b**) dose–effect (**c**) and temperature effect (**d**) and others respectively, in the cecum of broilers reared under normal or cyclic heat stress conditions. Dietary treatments were 0, 1, and 2% SPP in diets respectively. The temperature of the rooms was maintained at either thermoneutral (NT; 21 °C) or cyclic heat stress (CHS; 31 °C; 6 h daily) for seven days.

The effect of dietary SPP supplementation at NT and CHS of the thirty most abundant genus in the cecum of broiler chickens is presented in Fig. 5. Bacteroides were the most abundant bacteria at the genus level. Taxon-based analysis revealed that *Kineothrix* is the only genus that showed an interaction effect (P = 0.042). The genus *Bacillus* (P = 0.007) and *Saccharofermentans* (P = 0.002) showed dose–effect and were found to be higher in chickens supplemented with 1% SPP in diets in comparison to their counterparts. The abundance of genus *Bacteroides* (P = 0.036) and *Limosilactobacillus* (P = 0.038) were lower while that of *Alistipes* (P = 0.036), *Akkermansia* (P = 0.030), *Subdoligranulum* (P = 0.007) and *Pseudoflavonifractor* (P = 0.001) were higher in chickens exposed to CHS showing temperature effect.

## Discussion

The HS has devastating effects on various aspects of chicken. Parameters related to growth performances are severely affected when chickens are reared under HS conditions^2,3,14^. This is evident from the present study where the percent difference in body weight, ADG, and ADFI was decreased while FCR was increased when birds were reared under six hours of CHS for seven days. The reason behind this could be related to increased reactive oxygen species (ROS) generation and modulation of nutrient transporters^15,16^ and evident from enhanced NOX4 gene expression in CHS of this study. A decrease in the appetite due to the anorexic hormone secretion^17^ could be the other reason and can be correlated with the reduction in ADFI in the present study. Although, in the present study, the final weight of the chickens was similar among treatment groups and had no temperature effects. However, numerically lower body weight (P = 0.090) in CHS treatment indicates that the birds were subjected to lower severity of HS. Maintaining metabolic rate is important to control the heat production in the body. However, during HS the metabolic rate tends to be increased in broilers^18^. Under such circumstances, the chicken may stop feeding to regulate the increment in metabolic rates under CHS.

Supplementation of dietary SPP was done as a prebiotic to identify its role in growth performances under CHS. No interaction or dose effects on the growth performances of the birds could be due to the less severity of CHS in terms of temperature and duration. Furthermore, during the cyclic form of HS, the birds are brought back to the normal temperature after the selected period of HS each day. This makes the bird comfortable, returning to normal behavior. No mortality was reported throughout the study. Furthermore, non-significant higher ADG with increasing concentration of SPP supplementation in diets indicates its role in modulating growth performance and requires further exploration.

Body temperatures can be an effective indicator for identifying heat-tolerant chicken^19^. Due to the absence of sweat glands, chickens rely on respiration rates to maintain their body temperature^20^. Furthermore, dehydration is a common cause of high ambient temperature that results in increasing serum protein in chickens^21^. In the present study, CHS birds showed higher RT and plasma total protein in comparison to the NT. This also correlates with the previous studies where HS tends to enhance RT in chicken^19^.

HS has no temperature effect or dose effects on the digestive organ weight and length of chicken. Interaction effect has been seen in the weight of the duodenum in chickens. HS tends to decrease feed intake in broilers^2^. While prebiotics as a source of non-digestible fibers may enhance gut health by positively modifying the favorable bacteria in the intestine^9^. The interaction effects could be related to indigestible fibers present in the SPP-containing diets. Although there is a significant decrease in the ADFI in HS broilers, the adverse effects of HS could not be seen on the digestive organs in this study. It is well documented that the nature of HS plays an important role in creating thermo-balance and chicken partially acclimatizes to the higher temperatures in CHS compared to constant HS^22^. The possibility of developing thermotolerance may exist. Chicken reared under a cyclic form of HS may recover until the next day of heat exposure and the tendency to tolerate stress may increase after each day. During the acclimatization period, the chicken may show normal feeding behavior due to which the weight and length were not significantly affected.

The genes such as Zo1, Zo2, Claudin-1, and Occludin are known to possess a role in intestinal health by mediating gut integrity and permeability^23^. No significant variation in the expression of these genes indicated minimal SPP and CHS effects. The glucagon-like peptide is known to have a suppressive effect on food intake in chicken^24^. Higher expression of GLP-2 in the 2% of SPP supplemented diets might be due to its reducing action towards feed intake arising from the unfavorable taste. It has been previously suggested that the NOX4 gene is an effective marker for ROS generation^16^ and HSP70 helps in protecting the cells from the lethal effects of oxidative stress^25^. Enhanced expression of NOX4 gene in CHS exposed chicks indicates higher ROS generation depicting the adverse effect of rearing chickens at high ambient temperatures. Concomitantly, lower expression of SOD in the same treatment group (1% HS) confirms the beneficial effect of 1% SPP in diets. The enhanced expression of SOD in HS broilers along with its defensive role in eradicating superoxide anion free radicals has already been elucidated previously^26,27^.

Gut microbiota plays important role in modulating growth performance through intestinal health. High ambient temperature has been associated with modulation in the intestinal microbiota and dysbiosis in chickens^28^. The variation in alpha-diversity depends on many factors including the type, duration, and intensity of HS^29^. For instance, acute HS did not affect the alpha-diversity in the cecum when broilers were exposed to one-time HS at 31 ^◦^C for 6 h^11^. Contrary to this, the alpha-diversity in the ileum was modified by an enhanced number of observed species, Chao 1, and whole-tree phylogenetic diversity when broilers were exposed to chronic HS at 31 °C from 28 to 42 days^28^. In the present study, all the parameters related to alpha-diversity were similar and not affected. The discrepancy in the results in our study could be explained by the shorter HS exposure time (7 days) that was provided in the cyclic form (6 h daily).

Beta-diversity determined by unweighted and weighted UniFrac distances explains the presence or absence of observed organisms and their abundance in the sample. The HS is associated with intestinal injury via pathogenic permeability^2^. Significant differences in the beta-diversity of both unweighted and weighted UniFrac distances specify the modulation in the microbial presence under different temperature conditions.

In the present study, Firmicutes and Bacteroidetes were the major dominant phyla in the cecum, which is in correlation with the previous studies^11,30^. Contrary to this, Bacteroidetes and Firmicutes were the major abundant phyla in the cecum of laying hens^31^. This could be because several factors influence the gut microbiota including farm management, additives, and the age of the animal^32^. Furthermore, in the present study, CHS decreased Bacteroidetes and Candidatus Melainabacteria, a phylum related to Cyanobacteria in the cecum of broiler chickens. This correlates with the previous studies reporting a decrease in cecum Bacteroidetes and Cyanobacteria in the chickens exposed to HS for 14 days^30^. In another study, HS was found to increase the Firmicutes abundance and decreased Proteobacteria and Bacteroidetes in the ileum of broiler chickens^28^. In contrast, CHS had no significant effects on the relative abundance of Bacteroidetes in the cecum of laying hens^31^. This indicates that phylum microbiota varies following the breeds and digestive track location^32^. It has been suggested that Bacteroidetes encode carbohydrate-active enzymes in the gut^33^. The uptake of carbohydrates is adversely affected under HS ^16^. This indicates that a decrease in the phylum Bacteroidetes could be mediated through the reduced uptake of carbohydrates under HS conditions. In the present study, the abundance of Verrucomicrobia and other phylum bacteria was increased in CHS exposed chickens. This is in correlation with the previous study in rats, where the relative abundance of Verrucomicrobia was increased on the 7th day of HS^34^. The phylum Verrucomicrobia consists of the genus *Akkermansia* a mucin degrading bacteria^35^. HS enhances pathogen penetration and mucin degradation in chickens^29^. An increase in the genus *Akkermansia* from phylum Verrucomicrobia along with other bacterial phyla in this study could be associated with the degradation of mucin under HS resulting in an enhanced abundance of other bacteria that may act as a pathogen. An increase in the abundance of *Akkermansia* is associated with the reduction in body weight in HS rats^34^. In confirmation, the abundance of *Akkermansia* was increased in CHS chickens accompanied by a reduction in growth performance parameters such as percent difference in body weight, ADG, and ADFI.

The genus *Bacteroides* was found to be the most abundant bacteria in this study. This is in agreement with the earlier studies conducted on chickens^30,36^. *Bacteroides* are known to produce short-chain fatty acids and facilitate the breaking down of complex molecules to nurture the gut microbiota^37,38^. Decrease in the abundance of genus *Bacteroides* in CHS birds correlated with the previous studies^30^ and indicates the adverse effect of HS on the proliferation of microbiota in the gut.

The genus *Limosilactobacillus* is known to produce antimicrobials^39^ that may possess benefits to gut health by restricting the growth of pathogenic microorganisms in chickens. In a recent study, dietary supplementation of *Limosilactobacillus* has shown an inhibitory effect on the *Salmonella* activity in chicken^40^. The correlation of HS with pathogen penetration is already been established^16,28^. A decrease in the abundance of genus *Limosilactobacillus* in this study suggests that birds exposed to CHS are more prone to pathogenic invasions such as *Salmonella* due to the reduction in the production of antimicrobials in the gut.

*Alistipes* have been known as a relatively new genus of bacteria that possesses both beneficial as well as pathogenic activity^41^. Previous studies suggested that acute HS has no effect on the abundance of *Alistipes* but its number decreases in 2% SPP supplemented diets and explained its direct correlation with the phylum Bacteroidetes^11^. The results of the present study also indicated a similar decreasing pattern in CHS birds as the abundance of genus *Alistipes* that belong to phylum Bacteroidetes was significantly reduced when birds were exposed to 7 days of CHS.

The genus *Bacillus* is a spore-forming facultative anaerobe that fosters its beneficial effects on the gut by producing antimicrobial peptides to reduce infectious diseases in chickens^42,43^. The growth-promoting effects of various *Bacillus* strains have made it a popular probiotic product for its use in chicken feed^44^. Another bacterial genus *Saccharofermentans* ferment carbohydrates or starch to produce acetate, lactate, fumarate, and hydrogen peroxide as the end products^45^. Lactic acid and short-chain fatty acid could reduce the pH of the gut and then inhibits the growth of other bacteria, including enteropathogens^36^. An increase in the abundance of *Bacillus* and *Saccharofermentans* in a 1% SPP supplemented diet suggested its beneficial role in the gut for improving growth performances in chickens.

In conclusion, HS adversely affected the growth performance parameters in chickens. A decrease in the spleen and plasma total protein in CHS chickens signifies its immunosuppressive and dehydrating effects while enhanced intestinal health and stress-related gene expression (GLP-2 and NOX4) and pathogenic microbiota indicate challenged gut integrity and oxidative stress. Enhancement in the gut favorable bacteria in 1% SPP supplemented diets suggested its role in mitigating its adverse effects through modulating gut microbiota.

## Material and methods

### Animal and housing

This experiment was conducted at the research facility of the Gyeongsang National University, Korea. All the experimental procedures were approved by the Animal Ethics Committee of the Gyeongsang National University (GNU-200916-C0057) and complied with the latest version of the ARRIVE guidelines^46^. All the methods were performed following the relevant guideline and regulations.

A total of 180–1-week-old Ross 308 broiler chicks with similar body weight were distributed in three dietary treatment groups and two environmental conditions following a 3 × 2 factorial design. Birds were reared following the managemental guide of Ross 308 broiler chicks until 4 weeks of age and provided with ad libitum of feed and water. The feeding program was initialized on the 8th day of age. Birds were fed grower (day 8 to day 21) and finisher (day 22 to day 35) with three different experimental diets in each room containing 0, 1, and 2% shredded, steam-exploded pine particles passing through a 10-mesh sieve replacing corn in their feed ingredients (Supplementary Table 1) and has been published elsewhere^47^. On the 28th day of age, the CHS experiment was initialized after taking the body weight of each bird. Further, the birds were allotted to six treatment groups in two identical rooms and each treatment had six replicates of five birds per cage making it 30 birds per treatment. The temperature of the two rooms was either maintained at the recommended thermoneutral (NT) (21 °C) or gradually increased for providing CHS conditions (Supplementary Fig. 1). The heat treatment starts from 9:00 am with an increment of 2 °C/h to reach 31 °C within five hours and is maintained thereafter for one more hour before it was brought back to thermoneutral temperature to act as CHS. Birds were reared for seven days until the 35th day of age. Feed and water were provided ad libitum. Body weight was recorded before starting and after finishing the CHS experiment. Average daily feed intake (ADFI), Average daily gain (ADG), and feed conversion ratio (FCR) were calculated based on the data recorded before and after the experiment in each cage, and mortality was recorded daily if happened.

### Rectal temperature and sample collection

The rectal temperature (RT) was recorded using a digital thermometer (HI 91610, Hanna instruments Inc., Padova, Italy) by inserting the probe 3 cm inside the rectum before (29th day of age) and after (35th day of age) HS on the final day of CHS.

The intestinal length (duodenum, jejunum, ileum, and cecum) and weight of vestigial organs were recorded and expressed as cm per hundred gm body weight and percent body weight respectively. The whole cecum (both pouches) from each chick was first tied at the ileocecum junction, excised carefully to measure the length, and was then immediately snap-frozen in liquid nitrogen for microbiome studies.

After finishing the heat challenge experiment on the 35th day of age, seven birds from each group were humanly sacrificed to collect blood samples in a heparinized vacutainer for plasma processing as mentioned previously^47^. Briefly, plasma was harvested by centrifuging the blood samples at 2000×*g* at 4 °C for 10 min and was then immediately stored at – 20 °C until use.

The concentrations of glucose, total protein, triglycerides, and total cholesterol were determined in plasma using a VetTest Chemistry Analyzer (IDEXX Co., Ltd., Westbrook, ME, USA) with a dry-slide technology as described previously^47^. Briefly, the slides for the above-mentioned parameters were purchased (IDEXX Co., Ltd., Westbrook, ME, USA) and installed in the analyzer. The plasma sample was automatically loaded through an automated pipettor and the results generated were then recorded.

### Quantitative real-time PCR (qPCR)

Total RNA was extracted from the duodenum using Trizol™ reagent (Thermo Fisher Scientific, Massachusetts, Waltham, MA, USA) following the manufacturer's protocol. The concentration and purity of each sample were analyzed by using Nanodrop (Thermo Scientific, Massachusetts, Waltham, MA, USA). The samples were then reverse transcribed using the PrimeScript^TM^ first-strand cDNA synthesis kit (Takara, Tokyo, Japan) following the manufacturer’s guide. Differential expression of some gut health and stress-related genes (Supplementary Table 2) was performed using StepOnePlus™ real-time PCR systems (Life Technologies, California, Carlsbad, USA) according to the following protocol: 10 min at 95 °C, followed by 40 cycles of 15 s at 95 °C and 1 min at 60 °C. Each reaction consists of a total 20 μL volume containing Power SYBR™ green PCR master mix (Life Technologies, California, Carlsbad, USA), 10 pmol concentration of forward and reverse primer specific for each gene and cDNA. The geometric mean of Glyceraldehyde-3-phosphate dehydrogenase (GAPDH) and β-actin were used as housekeeping genes for normalization. Relative expression was determined using the 2^−ΔΔct^ algorithm.

### DNA extraction and sequencing

Total genomic DNA from cecal samples was extracted using DNeasyPowerSoil Kit (Qiagen, Hilden, Germany) following the manufacturer’s instructions. The quantification of the DNA samples was performed using Quant-IT PicoGreen (Invitrogen, Waltham, MA, USA). For metagenome estimation, a 16S metagenomic sequencing library was constructed using a Herculase II Fusion DNA Polymerase Nextera XT Index Kit V2 (Illumina, San Diego, CA, USA). The sequencing of the library was done with the Illumina platform at Macrogen, Inc. (Seoul, Korea). The primers used were Bakt_341F (CCTACGGGNGGCWGCAG) and Bakt_805R (GACTACHVGGGTATCTAATCC) targeting the V3 and V4 regions of the ribosomal RNA. FASTQ files for each sample were created for quality profiling, adapter trimming, and read filtering using the fastp program^48^. FLASH (v1.2.11) software was used to assemble paired-end reads into one sequence^49^, and sequences within 400–500 bp were only used. The number of operational taxonomic units (OTUs) was determined by de novo clustering with a 97% sequence identity cutoff using the CD-HIT-EST program^50^. BLAST + (v2.9.0) program was used to check each OTU sequence for its taxonomic similarity against the reference database (NCBI 16S Microbial)^51^. Identical coverage of less than 85% was identified as not defined. QIIME (v1.9) software was used to analyze microbial communities in terms of OTU abundance and taxonomic information. For the Alpha-diversity of a specific sample, species diversity and homogeneity among the microbial community were evaluated through Shannon, Goods Coverage, and Inverse Simpson Index. Beta-diversity was evaluated using unweighted/weighted UniFrac distances.

### Statistical analysis

The analysis was performed following a 3 × 2 factorial design. All the data related to growth performances and gene expression were analyzed using the general linear model (GLM) procedure of two-way ANOVA using IBM SPSS statistics software package 25. 0 (IBM software, Chicago, IL, USA). Each cage was treated as an experimental unit for growth studies while an individual bird was treated as the experimental unit for organ weight, length, and body temperatures. All the data were expressed as means ± SEM. Duncan's multiple range test was used if an interaction exists. Differences were considered statistically significant at P < 0. 05. For gene expression, orthogonal planned contrasts were performed following comparison using the “Contrast statement” of the SAS software. Alpha-diversity (community richness and diversity) and taxonomic analysis (phylum and genus) were analyzed using the scheirer-Ray-Hare test and the Dunn test was used when P values were significant. The “Rcompanion” package of the R software version 4.0.3 (R Core Team, 2020) was used. PERMANOVA was performed for analyzing beta-diversity.

### Ethics approval

All animal handling procedures complied with the Animal Ethics Committee at Gyeongsang National University (GNU-200916-C0057).

## Supplementary Information

Supplementary Information.

## Data Availability

The sequencing data of cecal microbiota is deposited into the Sequence Read Archive (SRA) database of NCBI and is available on BioProject Accession Number PRJNA835667.
