# Supplementary material for: Dietary shredded steam-exploded pine particle supplementation as a strategy to mitigate chronic cyclic heat stress by modulating gut microbiota in broilers
Source: Sci Rep. 2022 Nov 16;12:19704. doi: 10.1038/s41598-022-24031-w (PMC9669035; doi:10.1038/s41598-022-24031-w)
Supplement: Supplementary file 1 — Supplementary Information. [file 41598_2022_24031_MOESM1_ESM.docx]

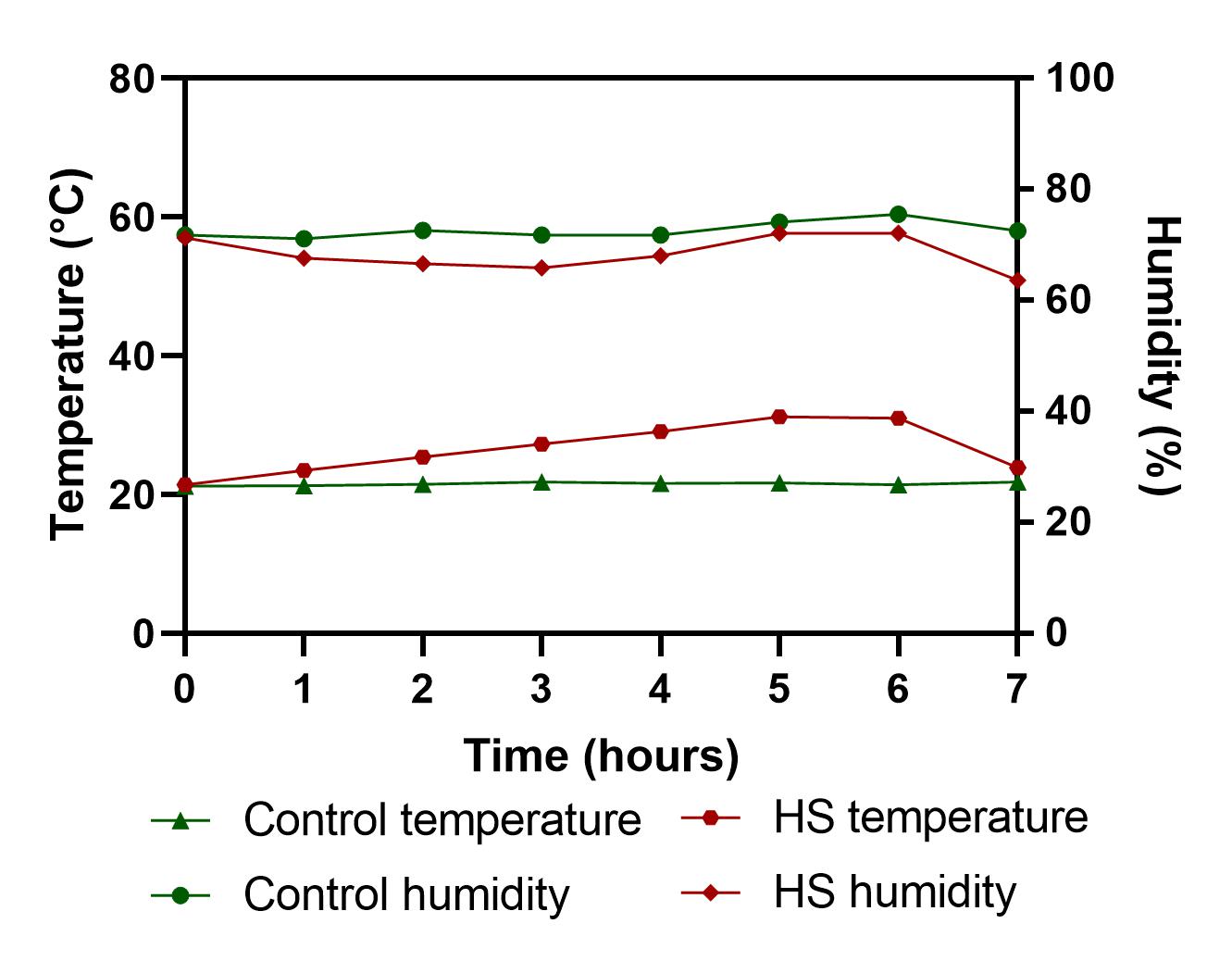


Supplementary Figure 1: The temperatures and humidity of thermoneutral and heat stress rooms maintained during the seven days of the experiment.

Supplementary Table 1: Formula and chemical composition of the experimental diets.

| Items | Grower | | | Finisher | | | |
| --- | --- | --- | --- | --- | --- | --- | --- |
|  | 0% | 1% | 2% | 0% | 1% | 2% |  |
| Ingredients (%) |  |  |  |  |  |  |  |
| Yellow corn, ground | 57.1 | 55.2 | 53.2 | 63.3 | 61.2 | 59.1 |  |
| Soybean meal | 33.3 | 33.5 | 33.8 | 28.7 | 29.0 | 29.3 |  |
| Corn gluten meal | 3.0 | 3.0 | 3.0 | 2.5 | 2.5 | 2.5 |  |
| Limestone | 1.6 | 1.6 | 1.5 | 1.5 | 1.5 | 1.5 |  |
| Tallow | 3.6 | 4.4 | 5.1 | 3.3 | 4.0 | 4.8 |  |
| Mono-dicalcium phosphate | 0.42 | 0.43 | 0.44 | 0.05 | 0.05 | 0.05 |  |
| Salt | 0.3 | 0.3 | 0.3 | 0.3 | 0.3 | 0.3 |  |
| Choline chloride (50%) | 0.07 | 0.07 | 0.07 | 0.03 | 0.03 | 0.03 |  |
| DL-methionine hydroxy analogue, 88% | 0.15 | 0.15 | 0.15 | 0.10 | 0.10 | 0.10 |  |
| L-Lysine | 0.17 | 0.16 | 0.15 | 0.04 | 0.05 | 0.05 |  |
| SPP | 0.0 | 1.0 | 2.0 | 0.0 | 1.0 | 2.0 |  |
| Vitamin mixture ^1^ | 0.2 | 0.2 | 0.2 | 0.2 | 0.2 | 0.2 |  |
| Mineral mixture ^2^ | 0.1 | 0.1 | 0.1 | 0.1 | 0.1 | 0.1 |  |
| Calculated values |  |  |  |  |  |  |  |
| TMEn, kcal/kg | 3103 | 3102 | 3102 | 3150 | 3150 | 3150 |  |
| Crude protein, % | 21.03 | 21.02 | 21.03 | 19.02 | 19.03 | 19.02 |  |
| Calcium, % | 0.91 | 0.92 | 0.91 | 0.82 | 0.81 | 0.82 |  |
| Available Phosphorus, % | 0.42 | 0.42 | 0.43 | 0.36 | 0.36 | 0.36 |  |
| Lysine, % | 1.15 | 1.15 | 1.15 | 1.01 | 1.01 | 1.01 |  |
| Methionine + Cystine, % | 0.84 | 0.83 | 0.83 | 0.71 | 0.72 | 0.71 |  |
| Analyzed values (%) |  |  |  |  |  |  |  |
| Moisture | 10.98 | 11.19 | 10.91 | 9.87 | 9.92 | 10.33 |  |
| Crude protein | 20.59 | 22.03 | 20.94 | 19.69 | 18.81 | 19.03 |  |
| Ether extract | 6.97 | 8.07 | 7.69 | 6.92 | 7.46 | 7.49 |  |
| Crude fiber | 2.70 | 3.12 | 4.11 | 2.77 | 3.43 | 4.13 |  |
| Ash | 4.32 | 4.34 | 4.31 | 4.80 | 3.87 | 3.72 |  |

^1^ Vitamin mixture provided the following nutrients per kg: vitamin A, 40,000,000 IU; vitamin D3, 8,000,000 IU; vitamin E, 10,000 IU; vitamin K3, 4000 mg; vitamin B1, 4000 mg; vitamin B2, 12,000 mg; vitamin B6, 6000 mg; vitamin B12, 20,000 µg; pantothenic acid, 20,000 mg; folic acid, 2000 mg; nicotinic acid, 60,000 mg. ^2^ Mineral mixture provided the following nutrients per kg: Fe, 30,000 mg; Zn, 25,000 mg; Mn, 20,000 mg; Co, 150 mg; Cu, 5000 mg; Ca, 250 mg; Se, 100 mg. Abbreviations: SPPs, shredded steam-exploded pine particles; TMEn, true metabolizable energy.

Supplementary Table 2: Primer sequences were used to evaluate the duodenum gene expression in broiler chickens.

| Gene ^1^ | Sequence | Accession number |
| --- | --- | --- |
| Zo1 | F: AAGTGGGAAGAATGCCAAAA | XM_015278981.2 |
|  | R: GGTCCTTGGATCCCGTATCT |  |
| Zo2 | F: GCCCAGCAGATGGATTACTT | XM_025144669 |
|  | R: TGGCCACTTTTCCACTTTTC |  |
| GLP-2 | F: CGTGCCACAGCCATTCTTA | NM_001163248.1 |
|  | R: AGCGGCTCTGCAAATGATTA |  |
| Claudin 1 | F: AAGGTGTACGACTCGCTGCT | NM_001013611.2 |
|  | R: CAGCAACAAACACACCAACC |  |
| Occludin | F: ACGGCAAAGCCAACATCTAC | NM_205128.1 |
|  | R: ATCCGCCACGTTCTTCAC |  |
| NOX4 | F: CCTCTGTGCTTGTACTGTGTAG | NM_001101829.1 |
|  | R: GACATTGGAGGGATGGCTTAT |  |
| HSP70 | F: GCTGAACAAGAGCATCAATCCA | AY143693.1 |
|  | R: CAGGAGCAGATCTTGCACATTT |  |
| SOD | F: AGGGGGTCATCCACTTCC | NM_205064.1 |
|  | R: CCCATTTGTGTTGTCTCCAA |  |
| GAPDH | F: TTGGCATTGTGGAGGGTCTTA | NM_204305.1 |
|  | R: GTGGACGCTGGGATGATGTT |  |
| Beta actin | F: ACCGGACTGTTACCAACA | NM_205518.1 |
|  | R: GACTGCTGCTGACACCTT |  |

^1^ Abbreviation: Zo1: Zonula occluden 1; Zo2: Zonula occluden 2; GLP-2: Glucagon-like peptide-2; NOX4; Nicotinamide adenine dinucleotide phosphate oxidase 4; HSP70: Heat shock protein 70; SOD: Superoxide dismutase; GAPDH: Glyceraldehyde-3-phosphate dehydrogenase.

Supplementary Table 3: Plain contrast analysis of the various genes

| Genes | Planned contrast | | | | | | | |
| --- | --- | --- | --- | --- | --- | --- | --- | --- |
|  | Zo1 | Zo2 | GLP-2 | Claudin-1 | Occludin | Nox4 | HSP70 | SOD |
| 0%NT vs 1%NT | NS | NS | NS | NS | NS | NS | NS | NS |
| 0%NT vs 2%NT | NS | NS | NS | 0.038 | NS | 0.026 | NS | NS |
| 0%NT vs 0%HS | NS | NS | NS | NS | NS | NS | NS | NS |
| 0%HS vs 1%HS | NS | NS | NS | NS | NS | NS | NS | 0.037 |
| 0%HS vs 2%HS | NS | NS | NS | NS | NS | 0.044 | NS | NS |
| 0%NT vs 1%HS | NS | NS | 0.047 | 0.027 | NS | 0.024 | 0.030 | NS |
| 0%NT vs 2%HS | NS | NS | NS | NS | NS | 0.001 | NS | NS |

NT: thermoneutral temperature; HS: heat stress; NS: not significant; Zo1: Zonula occluden 1; Zo2: Zonula occluden 2; GLP-2: Glucagon-like peptide-2; NOX4; Nicotinamide adenine dinucleotide phosphate oxidase 4; HSP70: Heat shock protein 70; SOD: Superoxide dismutase; GAPDH: Glyceraldehyde-3-phosphate dehydrogenase
